# NeuroPep: a comprehensive resource of neuropeptides

**DOI:** 10.1093/database/bav038

**Published:** 2015-04-29

**Authors:** Yan Wang, Mingxia Wang, Sanwen Yin, Richard Jang, Jian Wang, Zhidong Xue, Tao Xu

**Affiliations:** ^1^Key Laboratory of Molecular Biophysics of the Ministry of Education, College of Life Science and Technology, Huazhong University of Science and Technology, Wuhan, Hubei 430074, China, ^2^School of Software Engineering, Huazhong University of Science and Technology, Wuhan, Hubei 430074, China, ^3^Department of Computational Medicine and Bioinformatics, University of Michigan, Ann Arbor, MI 48109, USA and ^4^National Laboratory of Biomacromolecules, Institute of Biophysics, Chinese Academy of Sciences, Beijing 100101, China

## Abstract

Neuropeptides play a variety of roles in many physiological processes and serve as potential therapeutic targets for the treatment of some nervous-system disorders. In recent years, there has been a tremendous increase in the number of identified neuropeptides. Therefore, we have developed NeuroPep, a comprehensive resource of neuropeptides, which holds 5949 non-redundant neuropeptide entries originating from 493 organisms belonging to 65 neuropeptide families. In NeuroPep, the number of neuropeptides in invertebrates and vertebrates is 3455 and 2406, respectively. It is currently the most complete neuropeptide database. We extracted entries deposited in UniProt, the database (www.neuropeptides.nl) and NeuroPedia, and used text mining methods to retrieve entries from the MEDLINE abstracts and full text articles. All the entries in NeuroPep have been manually checked. 2069 of the 5949 (35%) neuropeptide sequences were collected from the scientific literature. Moreover, NeuroPep contains detailed annotations for each entry, including source organisms, tissue specificity, families, names, post-translational modifications, 3D structures (if available) and literature references. Information derived from these peptide sequences such as amino acid compositions, isoelectric points, molecular weight and other physicochemical properties of peptides are also provided. A quick search feature allows users to search the database with keywords such as sequence, name, family, etc., and an advanced search page helps users to combine queries with logical operators like AND/OR. In addition, user-friendly web tools like browsing, sequence alignment and mapping are also integrated into the NeuroPep database.

**Database URL**: http://isyslab.info/NeuroPep

## Introduction

Neuropeptides are small proteineous substances that are produced by neurons, released in a regulated fashion, and act on either neural substrates, such as neurons, glial cells or on non-neuronal target cells, such as a gland or muscle (1). It has been shown that different neuropeptides are involved in a number of physiological processes such as food intake, metabolism, stress control, pain perception, social behaviors, learning, memory, etc. (2–5). Neuropeptides are typically 3–100 amino-acid-residue long, produced from larger precursor molecules by a series of post-translational processing (6). There are two general approaches (‘function-first’ or ‘peptide-first’) to discover neuropeptides. The function-first approach is based on bioassays, receptor-binding assays and genetic analysis, while the peptide-first approach is based on cDNA cloning of precursor, using neuropeptide precursor processing enzymes, peptidomics, etc. (7). Since the first neuropeptide substance P was discovered by van Euler and Gaddum in 1931 (8) and sequenced in 1971 (9), there has been a tremendous increase in the number of identified neuropeptides over the last 40 years. Furthermore, due to their wide range of roles in health and disease, neuropeptides are considered as attractive therapeutic targets for nervous-system disorders such as depression, anxiety and Parkinson’s disease (10–12). Several databases like EROP-Moscow (13), SwePep (14), PeptideDB (15), PepBank (16) have been developed to hold general bioactive peptides. There are also some databases such as APD2 (17), CAMP (18), DAMPD (19), which were designed for specific antimicrobial peptides. However, there are only two recent databases specific for neuropeptides.

About 90 genes encoding classical and candidate neuropeptides in mammalian genomes were provided in the online neuropeptide database www.neuropeptides.nl (1). It provided genes, precursors and the processed active peptide names on the website and also provides hyperlinks to bioinformatics databases on genomes, transcripts, brain expression and homologies to other species. The NeuroPedia database was mainly designed for identification of neuropeptides using mass spectrometry data (20). It extracted 847 neuropeptide sequences of eight organisms belonging to the phylum Chordata and collected 3401 identified spectra from NIST spectral libraries and their own in-house spectral datasets (20). Since the two databases were specialized for the authors’ study, many invertebrate-specific neuropeptides were not considered such as allatostatins, pyrokinins, crustacean cardioactive peptides, pigment-dispersing factors, etc., and neither of the databases gave detailed annotations for each neuropeptide sequence. Although the invertebrate neuropeptide characterization lagged behind the vertebrate neuropeptide, there were many new invertebrate neuropeptides that were identified owing to the development of the mass spectrometry technique in recent years (21–23).

Therefore, this study focused on collecting as many known neuropeptide sequences and related information as possible and integrating them into a searchable archive. With this in mind, we not only extracted entries from the public resources of UnitProt (24), databases www.neuropeptides.nl and NeuroPedia, but also retrieved neuropeptide sequences in the abstracts and full texts of literatures in MEDLINE by text-mining methods. To better serve the community, the database also provided comprehensive information for each entry and integrates user-friendly browsing and search facilities along with useful tools such as BLAST (25), ClustalW (26) and Map. We hope that this database will be helpful for the research community and be a valuable resource for neuropeptide-based therapeutics development.

## Materials and methods

### Data collection and compilation

NeuroPep database is a comprehensive resource of neuropeptides. The data were collected from various resources including MEDLINE abstracts, full papers, UniProt, databases www.neuropeptides.nl (1) and NeuroPedia (20). First, we searched Pubmed with the keyword ‘neuropeptide’ which returned 240 388 articles of which only 7277 contained peptide sequences. The Peptide::Pubmed (16) method was used to extract the peptide sequences from the abstracts of these articles. In total, 10 515 peptide sequences were extracted from the 7277 different abstracts. To check whether or not the sequence extracted from the abstract is a neuropeptide, we extracted the peptide name or the term describing the peptide sequence and then compared it with a neuropeptide name list which was built based on www.neuropeptides.nl and UniProt. It was found that many of these sequences are analogs, antagonists, agonists, fragments, motifs, etc. After filtering, 2595 natural neuropeptide sequences remained. Meanwhile, we also mined name, family, modification and organism information from the corresponding texts.

Furthermore, we wrote a perl script to select articles which may contain the neuropeptide sequences identified by MS/MS in their full texts. The script was designed for the keyword search by ‘Neuropeptide’ and ‘Mass Spectrometry’ from the title/abstract fields and returned 579 articles out of the 240 388 articles. Neuropeptides described in the full text articles were also selected based on the family or peptide names which were mentioned in the articles. 3471 sequences along with their name, family and organism information were collected manually from the full texts. In addition, neuropeptide precursors were selected from the entries of UniProt (release 2013-05) which were annotated as ‘neuropeptide’ in the ‘keyword’ line. 2715 neuropeptide sequences annotated as ‘peptide’ or ‘chain’ in the ‘Feature’ line were retrieved from these precursors. Moreover, we downloaded the corresponding UniProt protein file according to the UniProt ID from the blink results of all the neuropeptide precursors listed in the database www.neuropeptides.nl. 2179 sequences were further retrieved from these protein files. Combined with all the 847 sequences collected from NeuroPedia database, the integrated dataset contained 11 807 sequences. During the process to remove redundancy according to the sequence and organism, we preferred to keep the UniProt entries. As a result, 5949 non-redundant entries remained.

### NeuroPep server and web interface

**Figure 1.**
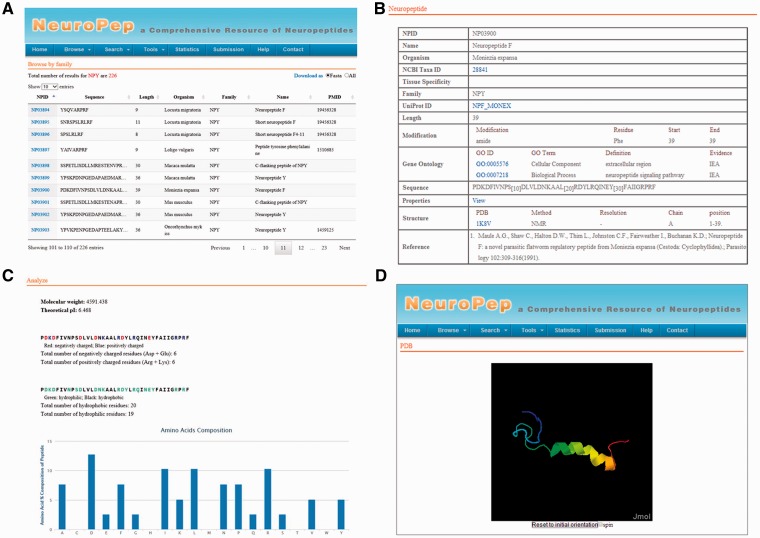
An overview of the user interface of NeuroPep. (**A**) The browse output of NPY neuropeptide family. (**B**) An example of an entry NP03900 of the NPY family. (**C**) The properties page of the entry NP03900. (**D**) The structure view page of the entry NP03900.

We constructed the NeuroPep server using Apache Tomcat Server 7 with MySQL Community Server 5.6. HTML, JSP and Ajax were used to build the front-end, and Java and JDBC were used to implement the web services and data management. The Highcharts Javascript package was used to add interactive plots to the web site. An overview of the user interface of NeuroPep database is shown in Figure 1.

### Data organization

The data in NeuroPep database are organized into the following fields (Figure 1B):

Accession number: A unique identifier to tag each database entry. Each accession number begins with the characters ‘NP’ followed by five digits.
Name: The name of the neuropeptide which was collected from the literature or UniProt.
Organism: The scientific name of the organism with the neuropeptide sequence.
Tissue Specificity: The specific tissue expressing of the neuropeptide.
Family: The classification of the neuropeptide. It was extracted from the ‘SIMILARITY’ line of UniProt or literatures. In addition, entries without family information and neuropeptide-like proteins in phylum Nematoda were annotated as ‘NA’.
UniProt ID: the accession number in UniProt (if available) and a link to its UniProt entry.
Modification: The type of post-translational modification and the position in the sequence for each modified residue.
Sequence: The amino acid sequence of the peptide.
Structure: The three-dimensional structure of the neuropeptide from the PDB database. Jmol was integrated into the database to facilitate structure vision.
Reference: The source of the neuropeptide sequence. External links to the abstracts of the articles are also provided.

In addition to the above directly extracted information, we computed the frequency of each of the 20 standard amino acid types, isoelectric points and molecular weight of each neuropeptide. We also computed the frequency of each special type of amino acid (positive charge, negative charge, hydrophilic and hydrophobic) to help user to know which type of amino acid each neuropeptide prefers. The above information is shown in the field of properties (Figure 1C).

### Data retrieval

A powerful browsing facility allows a user to browse the database using three different major categories including neuropeptide family, organism and modification. There are 65 neuropeptide families and 493 organisms in the current database, and they are presented in alphabetical order. The modification field includes five most common modifications occurring in neuropeptide sequences including amidation, acetylation, pyroglutamination, sulfation and phosphorylation. The browse output has the option to sort the data by clicking on the column title. The sequences can be downloaded in fasta format, and all the information can be downloaded in txt format.

Furthermore, users can query the database by two types of search tools: quick search and advanced search. Quick search enables users to search the database by the following fields: NPID, organism, family, name, UniProt ID, sequence and PMID. When the sequence field is chosen, all the neuropeptide sequences containing the input sequence will be returned. Advanced search allows users to build complex search queries using logical conditions like AND/OR. In addition, advanced search allows users to specify a search range of the fields including length, molecular weight and isoelectric point.

### Integration of web tools

To facilitate analysis of neuropeptide sequences, various web-based tools have been integrated into NeuroPep. The following is a brief description of these tools.

*Blast*: To find sequences in NeuroPep that are similar to a user-provided sequence, we have incorporated the BLAST search tool on the website. It allows users to submit the sequence in FASTA format and choose the user-defined parameters including *E*-value cutoff, and the substitution matrix for sequence alignment. The output is shown in the standard BLAST output, which includes the matching sequences, BLAST score and *E*-value.
*ClustalW*: ClustalW, one of the most commonly used tools for multiple sequence alignment, is also integrated into the database to help users find the conserved motifs in a group of neuropeptide sequences. The input consists of multiple peptide sequences in FASTA format, and the output is the standard ClustalW multiple sequence alignment format.
*Map*: Given the sequence of a neuropeptide precursor, one may need to find all possible processed neuropeptides in the database. To facilitate this task, we have developed the Map tool. Map finds all peptides in the database that match exactly to a substring in the user-provided sequence. For each output sequence, the starting position in the input sequence where the matching substring begins is also provided.

### Submission

Users can submit their published or newly discovered data into NeuroPep via the online submission form. The NeuroPep server will update the database automatically once the newly submitted entry is confirmed by us. A confirmation email will be sent to the submitter after validation. Periodically, we will also search for and submit new entries from the newly published literatures and UniProt.

## Results and discussion

After removing redundancy and manual checking, the current release of the database (version 1.0, 2014-11-26) holds 5949 non-redundant entries, among which 4035 neuropeptide sequences have been confirmed by at least one reference. The 5949 non-redundant entries cover 493 organisms belonging to 65 different neuropeptide families. The 493 organisms can be classified into nine phyla including Annelida, Arthropoda, Chordata, Cnidaria, Echinodermata, Hemichordata, Mollusca, Nematoda and Platyhelminthes. Table 1 lists all the 65 neuropeptide families together with the number of different sources of neuropeptides and the phyla distribution of each family.

**Table 1.** The number of neuropeptides from each data source and phyla distribution for the 65 neuropeptide families in NeuroPep

Family name	Source			Phyla
	Lit^a^	SP^b^	NL^c^	
7B2	1	10	8	Chordata
ACBP	0	0	19	Chordata
Adrenomedullin	0	0	15	Chordata
AKH/HRTH/RPCH	69	113	0	Arthropoda
Allatostatin	275	86	0	Arthropoda; Nematoda
Apelin	0	0	16	Chordata
Arthropod CHH/MIH/ GIH/VIH hormone	68	49	0	Arthropoda
Arthropod PDH	33	4	0	Arthropoda
Bombesin/neuromedin-B/ranatensin	11	0	22	Chordata
Bradykinin	9	0	7	Chordata
Calcitonin	4	0	39	Chordata
Calcitonin-like peptide	6	0	0	Arthropoda
CART	2	10	7	Chordata
CCAP	10	6	0	Arthropoda; Mollusca
Cerebellin	4	0	20	Chordata
Chromogranin/secretogranin	21	0	77	Chordata
Corazonin	17	46	0	Arthropoda; Mollusca
Cystatin	0	0	22	Chordata
Ecdysis triggering hormone	7	0	0	Arthropoda
Endothelin/sarafotoxin	0	29	1	Chordata
FMRFamide related peptide	455	406	6	Arthropoda; Mollusca; Nematoda; Annelida; Platyhelminthes; Chordata; Cnidaria
Galanin	7	19	4	Chordata
Gastrin/ cholecysto kinin	35	86	112	Arthropoda; Chordata
Glucagon	14	0	228	Chordata
GnRH	32	0	42	Chordata; Mollusca
Insulin	9	4	194	Chordata; Mollusca; Arthropoda
Kinin	20	19	1	Arthropoda; Chordata
KISS1	3	0	14	Chordata
Leptin	0	0	21	Chordata
LWamide neuropeptide	3	23	0	Cnidaria
Melanin-concentrating hormone	3	33	4	Chordata
Molluscan ELH	0	43	0	Mollusca
Motilin	1	42	2	Chordata
Myomodulin	2	18	0	Annelida; Mollusca
Myosuppressin	23	20	0	Arthropoda
NAPRTase	0	0	4	Chordata
Natriuretic peptide	1	0	83	Chordata
Neurexophilin	0	0	16	Chordata
Neuromedins	6	7	2	Chordata
Neuropeptide B/W	1	11	0	Chordata
Neuropeptide S	0	4	0	Chordata
Neurotensin	15	0	21	Chordata
NPY	100	93	33	Arthropoda; Chordata; Mollusca; Platyhel minthes
Nucleobindin	0	0	11	Chordata
Opioid	56	117	2	Annelida; Chordata; Mollusca
Orcokinin	172	19	0	Arthropoda
Orexin	0	11	0	Chordata
Parathyroid hormone	0	4	19	Chordata
Periviscerokinin	19	263	0	Annelida; Arthropoda
POMC	3	0	204	Chordata
ProSAAS	1	27	0	Chordata
Pyrokinin	56	210	0	Arthropoda
Resistin/FIZZ	0	0	7	Chordata
RFamide neuropeptide	0	5	0	Chordata
Sauvagine/corticotropin-releasing factor/urotensin I	5	0	20	Chordata; Arthropoda
SCP	2	5	0	Mollusca
Serpin	17	0	87	Chordata
Somastostatin	22	0	33	Chordata
Somatotropin/prolactin	0	0	101	Chordata
Tachykinin	84	185	0	Annelida; Arthropoda; Chordata; Mollusca
TRH	1	0	17	Chordata
Urotensin-2	4	0	8	Chordata
Vasopressin/oxytocin	7	1	77	Arthropoda; Chordata; Mollusca
VGF	6	0	21	Chordata
YGGW-amide related peptide	0	14	0	Nematoda

ACBP, Acyl-CoA-binding protein; AKH/HRTH/RPCH, Adipokinetic hormone/ Hypertrehalosaemic factor / Red pigment-concentrating hormone; CHH/MIH/GIH/VIH, Crustacean hyperglycemic hormones/Molt-inhibiting hormone/Gonad-inhibiting hormone/ Vitellogenesis-inhibiting hormone; PDH, Pigment-dispersing hormone; CCAP, Crustacean cardioactive peptide; ELH, Egg-laying hormone; SCP, Small cardioactive peptide; CART, Cocaine-amphetamine-regulated transcript protein; POMC, Pro-opiomelanocortin; TRH, Thyrotropin-releasing hormone.

^a^Lit denotes literature.

^b^SP denotes UniProt.

^c^NL denotes the database www.neuropeptides.nl and NeuroPedia.

### Source distribution of neuropeptide families

The entries of NeuroPep were derived primarily from two types of sources. The first consists of public databases including UniProt, www.neuropeptides.nl and NeuroPedia. The second consists of scientific literature sources including MEDLINE abstracts and full texts. 3880 sequences were collected from the public repositories (UniProt:2216; databases www.neuropeptides.nl and NeuroPedia:1664). All 1664 neuropeptide sequences from www.neuropeptides.nl and NeuroPedia have UniProt entries, but they were not annotated as ‘neuropeptide’ in the keyword line of UniProt like the classical neuropeptides cerebellins, calcitonins, somastostatins, GnRHs, etc. To check whether the 2069 sequences from the literature have UniProt entries that lack the ‘neuropeptide’ annotation, these sequences were compared with all the ‘peptides’ and ‘chains’ in UniProt. This resulted in 117 such sequences. Therefore, the UniProt ID of each of these sequences was added to NeuroPep. As can be seen from Table 1, there are three families including calcitonin-like peptide, ecdysis triggering hormone, orcokinin, for which >90% of the entries were collected from the literature. All the 13 members of the neuropeptide family of calcitonin-like peptide and ecdysis triggering hormone were extracted from scientific literature. The orcokinin family contains 191 entries covering 16 organisms. Only five entries of Apis mellifera, two of Orconectes limosus, six of Procambarus clarkia and six of Rhodnius prolixus were collected from UniProt, the other 172 entries of 13 different organisms were collected from literatures. For another 45 neuropeptide families, 1–89% of the entries were obtained from text mining. For the remaining 17 neuropeptide families, all the entries were retrieved from the public resources.

### Phyla distribution of neuropeptide families

Fifteen out of all 65 neuropeptide families contain peptides from at least two different phyla. The most widely distributed FMRFamide-related peptide family spans seven phyla including Annelida, Arthropoda, Chordata, Cnidaria, Mollusca, Nematoda and Platyhelminthes. The other 50 neuropeptide families in the current version of the database are restricted to a single phylum. Eight families including pyrokinin, orcokinin, arthropod PDH, etc. are Arthropoda specific. There are 38 neuropeptide families like bradykinin, calcitonin, cerebellin, somatostatin, etc. which are only found in Chordata. All the egg-laying hormones and small cardioactive peptides are identified from Mollusca, and all the LWamide neuropeptides and all the YGGW-amide-related peptides are from Cnidaria and Nematoda, respectively.

### Neuropeptide and family distribution among phyla

**Figure 2.**
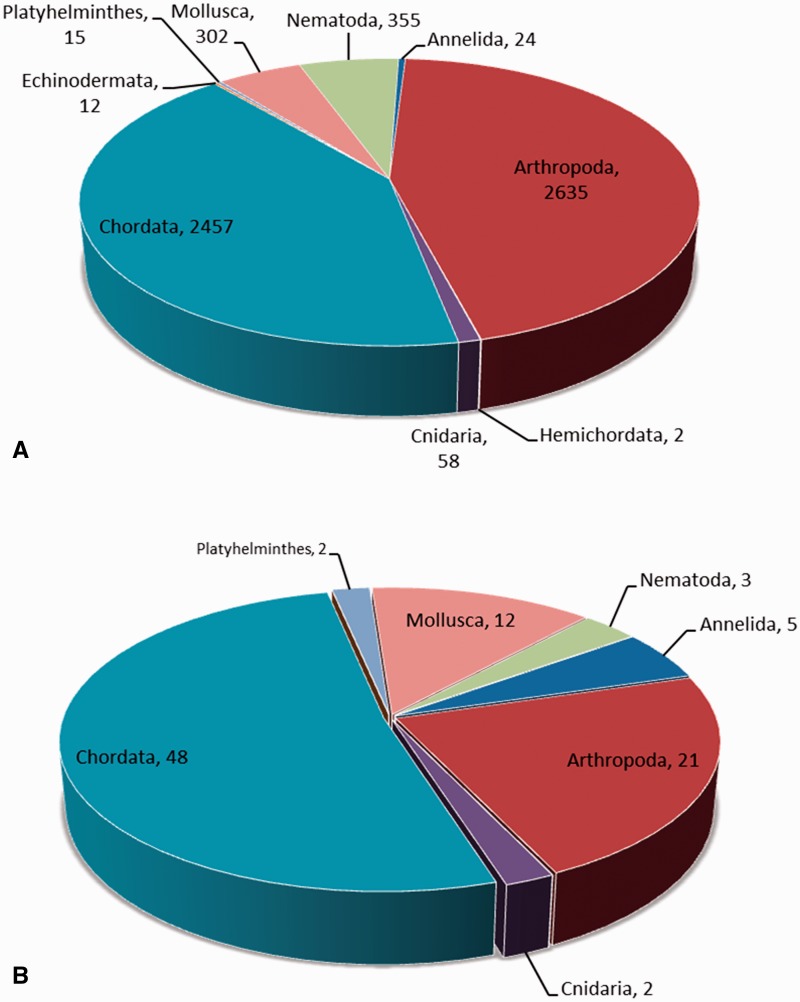
(**A**) The neuropeptide frequency distribution based on phyla. (**B**) The neuropeptide family distribution based on phyla.

The neuropeptide frequency distribution among phyla is given in Figure 2A. More than 44% (2635) neuropeptides are found in the phylum Arthropoda. The phylum Chordata has the second largest number of neuropeptides (2457, 41%). The majority of neuropeptides in Arthropoda and Chordata are found in the organisms of *Callinectes sapidus* (170) and *Rattus norvegicus* (294), respectively. The organisms with the most identified neuropeptides in the remaining phyla are as follows: *Caenorhabditis elegans* (Nematoda, 226), *Aplysia californica* (Mollusca, 150), *Anthopleura elegantissima* (Cnidaria, 18), *Eisenia foetida* (Annelida, 7), *Fasciola hepatica* (Platyhelminthes, 5), *Strongylocentrotus purpuratus* (Echinodermata, 8) and *Saccoglossus kowalevskii* (Hemichordata, 2).

Figure 2B shows the number of families for the neuropeptides in each phylum. The chordate phylum contains the highest number of neuropeptide families, which is 48, followed by Arthropoda and Mollusca, which are 21 and 12, respectively. Comparing Figure 2A and B, it is observed that the *Arthropoda phylum* has more identified neuropeptides than the *Chordata phylum*, but Chordata has more families.

### Amino acid and neuropeptide length distribution

**Figure 3.**
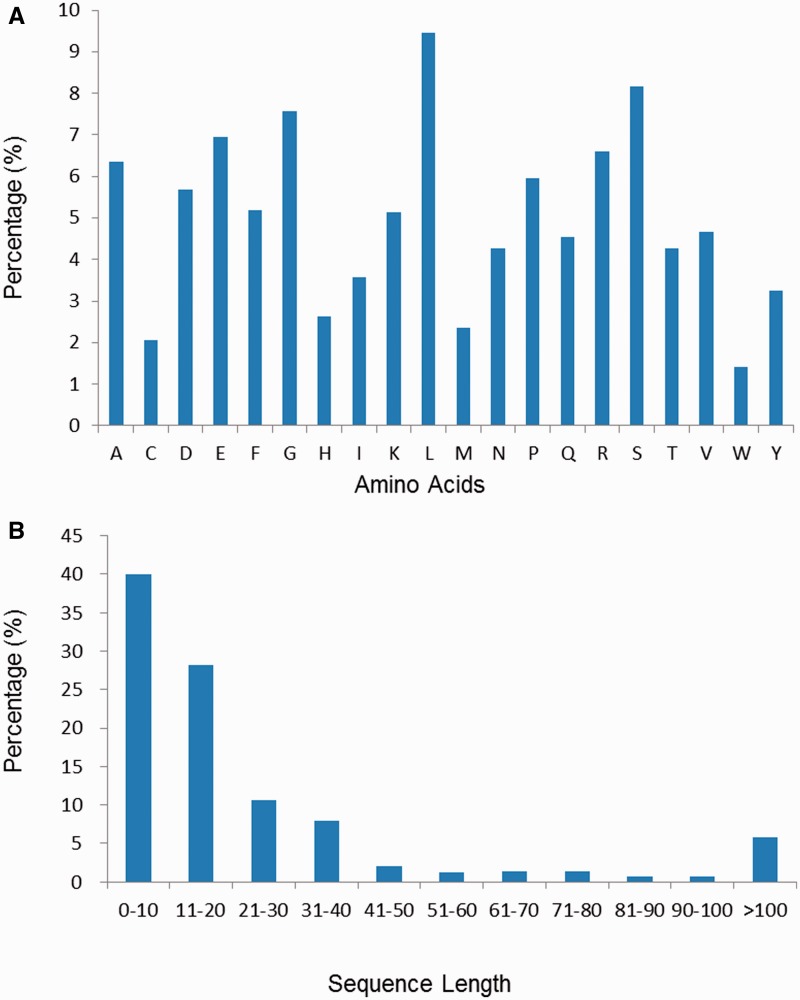
(**A**) The amino acid composition distribution of neuropeptides in NeuroPep database. (**B**) The amino acid length distribution of neuropeptides in NeuroPep database.

The amino acid composition and length distribution of neuropeptides of the current database are shown in Figure 3A and B, respectively. It is observed that residues like Leu, Ala, Ser, Glu and Gly are more abundant while residues like Trp, Cys, Met, His and Tyr are the least abundant. We have also computed separately the amino acid composition of invertebrate and vertebrate neuropeptides in the NeuroPep database. It is found that the Phe abundance in invertebrate neuropeptides is 2.6 times of that in vertebrate neuropeptides, while the Lys abundance in vertebrate neuropeptides is 3 times of that in invertebrate neuropeptides (Supplementary Figure S1). As shown in Figure 3B, the length of the majority of the neuropeptides (79%) is <30. There are 18 entries in the database with the shortest length of three-amino-acids long. A small portion (6%) of sequences are >100 amino acids in length, most of which (341/346) are annotated as ‘CHAIN’ in the feature line of UniProt.

### Comparison to other existing neuropeptide databases

We compared our database, NeuroPep, to the other two specific neuropeptide databases: the database at www.neuropeptides.nl (1) and the database NeuroPedia (20).

The developers of the database www.neuropeptides.nl mainly traced back the origin and the development of the concept of neuropeptide and proposed a conservative definition of neuropeptides. Based on the definition, they collected and analyzed over 90 genes encoding classical or candidate neuropeptides of the mammalian genomes. NeuroPedia was mainly designed for identification of neuropeptides using mass spectrometry data. It offered 847 neuropeptide sequences and the corresponding spectra data along with spectral library search tools.

Comparing to the above two neuropeptide database, NeuroPep has more experiment-validated neuropeptide sequences and cover more neuropeptide families and organisms. The current version of our database contains 5949 neuropeptide sequences belonging to 65 different neuropeptide families and covering 493 different organisms of nine different phyla. There are 16 invertebrate neuropeptide families such as allatostatin, periviscerokinin, orcokinin, pyrokinin, etc. that were not included by the databases www.neuropeptides.nl and NeuroPedia, which accounts for 28% of the NeuroPep database. The members of these 16 families are from Arthropoda, Mollusca, Nematoda, Annelida or Cnidaria. Note that 35% of the entries of our database were collected from text mining of MEDLINE abstracts and full text articles, which have not yet been collected or annotated by other public resources. Moreover, NeuroPep offers comprehensive annotations for each entry and user-friendly search facilities. Third, powerful analysis tools including BLAST, CLUSTALW and Map are also integrated in our database. Therefore, we think the NeuroPep database will be a useful resource for the research community.

## Summary and future perspectives

NeuroPep is a comprehensive neuropeptide database, which holds 5949 neuropeptide sequences originating from 493 organisms of nine different phyla. It is the most complete specific neuropeptide database to date. The database offers a user-friendly interface coupled with powerful browsing, searching and analysis tools. It allows users to submit new entries online. Each new entry will be validated before incorporating it into NeuroPep. It is very time-consuming to manually check the peptide sequences and extract related information from science papers. Therefore, a new text mining tool based on natural language processing is currently under development. It will be integrated into NeuroPep to update the database automatically as soon as novel neuropeptides become available.

## Supplementary Data

Supplementary data are available at *Database* Online.
